# Effectiveness of Oral Nifedipine Versus Intravenous Labetalol in Controlling Hypertension in Severe Preeclampsia: A Comparative Study

**DOI:** 10.7759/cureus.85899

**Published:** 2025-06-13

**Authors:** Maitri S Shah, Manvi Verma, Sushil Kumar, Himani Jivani

**Affiliations:** 1 Department of Obstetrics and Gynecology, MGM Medical College and Hospital, MGM Institute of Health Sciences, Navi Mumbai, IND

**Keywords:** hypertension in pregnancy, hypertensive emergency, intravenous labetalol, oral nifedipine, severe pre-eclampsia

## Abstract

Background: This study aimed to study and compare the efficacy and side effects between oral nifedipine and intravenous labetalol in controlling hypertension in acute cases of severe preeclampsia.

Methods: A prospective comparative study of pregnant patients with blood pressure >160/110 mm Hg after 20 weeks of gestation was conducted in the Department of Obstetrics and Gynecology of MGM Medical College and Hospital, Navi Mumbai, India, over a period of one and a half years from August 1, 2022, to December 31, 2023. A total of 100 cases were studied, 50 receiving oral nifedipine and the other 50 receiving intravenous labetalol.

Results: Though intravenous labetalol and oral nifedipine both are equally efficacious first-line anti-hypertensive drugs, oral nifedipine was observed to have a quicker onset of action, with a lesser number of doses required to reach the target blood pressure, with a convenient route of administration. Thus, it can be preferred in settings where intravenous access is challenging, remote or low-resource settings, or patients who prefer oral medication. Additionally, nifedipine works as a uterine relaxant, shown to increase urine output, has no effect on pulmonary vasculature, has fewer side effects, and costs 0.5% of the overall cost for intravenous labetalol.

Conclusion: Oral nifedipine could be a better choice between the two for treating hypertensive emergencies; however, the decision should be individualized, considering patient-specific factors, clinical setting, and resource availability.

## Introduction

Hypertensive disorders of pregnancy (HDPs), encompassing chronic hypertension (with or without superimposed preeclampsia/eclampsia), gestational hypertension, preeclampsia (with or without severe features), HELLP (hemolysis, elevated liver enzymes, and low platelet count) syndrome, and eclampsia, pose substantial risks of morbidity to both maternal and fetal health [1]. The prevalence of HDPs in India is reported to be 11% [2]. Severe hypertension in pregnancy is defined as a sustained systolic blood pressure of more than 160 mm Hg or a diastolic blood pressure of more than 110 mm Hg, necessitating urgent hospital evaluation [3].

HDPs are associated with adverse obstetric outcomes, including placental abruption, postpartum hemorrhage, prematurity, and fetal growth restriction. Several risk factors have been identified, such as advanced maternal age, a family history of HDP, a body mass index (BMI) exceeding 30 kg/m^2^, hyperglycemia, in vitro fertilization, and polycystic ovary syndrome [4]. The immediate management of severe hypertension in pregnancy focuses on blood pressure control, with hydralazine, nifedipine, and labetalol being commonly employed pharmacologic agents [5]. The current study aimed to compare the efficacy of oral nifedipine versus intravenous labetalol in managing hypertension in severe preeclampsia. This included pregnant patients with blood pressure >160/110 mm Hg after 20 weeks of gestation. This study also sought to evaluate associated complications, feto-maternal outcomes, and cost-effectiveness.

## Materials and methods

Study design and setting

This prospective comparative single-blind study investigated pregnant patients exhibiting blood pressure exceeding 160/110 mm Hg after 20 weeks of gestation. The research was conducted in the Department of Obstetrics and Gynecology of MGM Medical College and Hospital, Navi Mumbai, India, over an 18-month period, commencing on August 1, 2022, and concluding on December 31, 2023.

Sampling and sample size determination

A total of 100 hypertensive pregnant women with blood pressure greater than 160/110 mm Hg were systematically allocated into two groups: Group A, comprising 50 participants, received oral nifedipine, while Group B, also with 50 participants, received intravenous labetalol.

The sample size (N) was calculated using the formula $N = \frac{Z^2 \cdot p \cdot (1-p)}{D^2}$ where p is the incidence (0.10) [2], Z is equal to 1.96 (constant for 5% precision), and D is equal to 0.061 (allowable error of 6.1%). This yielded an initial sample size of approximately 93. To account for potential defaulters, a total sample size of 100 patients was ultimately selected.

Methodology

Following ethical approval from the Institutional Ethics Committee of MGM Medical College (approval number: DHR-EC/2022/SC/07/52), eligible subjects were recruited after providing written informed consent. Data pertinent to the study were systematically collected using a pre-tested, structured proforma.

Inclusion criteria

Pregnant women with blood pressure exceeding 160/110 mm Hg at greater than 20 weeks of gestation, with or without proteinuria, who provided written informed consent, were included in the study.

Exclusion criteria

Participants with pre-existing chronic hypertension, asthma, cardiogenic shock, cardiac failure, pulmonary edema, chronic obstructive pulmonary disease, renal disease, or bradycardia were excluded. Additionally, individuals who had received either nifedipine or labetalol within the preceding 24 hours, those with absolute contraindications to either medication, or those unwilling to provide written consent were also excluded.

Data collection and intervention

Each patient underwent a detailed history, comprehensive clinical examination, and a battery of laboratory investigations. Routine hematological, biochemical, and radiological tests were performed, including complete blood count (CBC), liver function tests (LFT), renal function tests (RFT), blood grouping (BG), prothrombin time-international normalized ratio (PT-INR), serology, thyroid-stimulating hormone (TSH), oral glucose challenge test (OGCT), and ultrasonography. Urine output and vital signs were continuously monitored. Funduscopic examination, cardiotocography, and ultrasound scans were also conducted to assess fetal well-being.

Patients were stratified into two intervention groups, Group A (oral nifedipine) and Group B (intravenous labetalol), each comprising 50 patients. Oral nifedipine was administered in a semi-recumbent position, up to a maximum of five doses. Ten milligram tablets were given at 20-minute intervals up to a maximum of five doses till the achievement of a target blood pressure of less than 150/100 mm Hg. Intravenous labetalol was administered incrementally in doses of 20 mg, 40 mg, 80 mg, 80 mg, and 80 mg, with 20-minute intervals between doses, until a target blood pressure of less than 150/100 mm Hg was achieved. Subsequent anti-hypertensive agents were prescribed based on individual patient requirements. Patients who were administered oral nifedipine were continued on the same regimen subsequently, and patients who were given intravenous labetalol were maintained on oral labetalol as per requirement. 

Statistical analysis

All collected data were meticulously entered into Microsoft Excel (Microsoft Corporation, Redmond, Washington, United States) and subsequently analyzed using IBM SPSS Statistics for Windows, Version 25.0 (IBM Corp., Armonk, New York, United States). Quantitative variables were presented as the mean value±standard deviation or as the median±interquartile range, as appropriate. Qualitative data were expressed as percentages and proportions. Appropriate statistical tests were employed to determine associations between variables, with a p-value of less than 0.05 considered indicative of statistical significance.

## Results

Demographic and baseline characteristics

Across both study groups, demographic and baseline clinical parameters exhibited comparability. In Group A (total 50 patients), 25 patients were aged 18-25 years, with another 25 patients in the 26-37-year age bracket. Group B (total 50 patients) comprised 26 patients aged 18-25 years and 24 patients aged 26-37 years. The observed age distribution difference between the groups was not statistically significant (p=0.84).

Gravidity distribution also showed no significant difference between the groups. Group A included 28 primigravida and 22 multigravida patients, while Group B had 30 primigravida and 20 multigravida patients. This difference was not statistically significant (p=0.68). The demographic characteristics have been tabulated in Table 1.

**Table 1:** Demographic characteristics of Group A (oral nifedipine) and Group B (intravenous labetalol) *p-value <0.05 statistically significant; chi-squared test applied

Variable	Groups		ꭓ^2^	P-value*
	Group A (%)	Group B (%)		
Age				
18-25 years	25 (50)	26 (52)	0.04	0.84
26-37 years	25 (50)	24 (48)		
Parity				
Primigravida	28 (56)	30 (60)	0.16	0.68
Multigravida	22 (44)	20 (40)		
Total	50 (100)	50 (100)	-	-

Upon admission, vital parameters, including systolic blood pressure, diastolic blood pressure, pulse rate, hemoglobin levels, and serum creatinine levels, were similar across both Group A and Group B. No statistically significant differences were detected in these parameters between the two groups (p>0.05).

Efficacy

The efficacy of the interventions was assessed by comparing the number of doses required to achieve blood pressure control and the time taken to reach the target blood pressure.

Table 2 illustrates the comparison of the mean number of doses administered. Group A, receiving oral nifedipine, required a mean of 2.28±1.05 doses to control blood pressure. In contrast, Group B, treated with intravenous labetalol, required a mean of 2.74±1.04 doses. This difference in the mean number of doses between the two groups was statistically significant (p=0.03), indicating that oral nifedipine achieved blood pressure control with fewer doses.

**Table 2:** Number of doses required and time taken to control blood pressure in minutes between Group A (oral nifedipine) and Group B (intravenous labetalol) *p-value <0.05 statistically significant; independent samples t-test applied

Variable	Groups				Test statistics	P-value*
	Group A		Group B			
	Mean	SD	Mean	SD		
Number of doses required	2.28	1.05	2.74	1.04	- 2.19	0.03
Time taken to control (in minutes)	45.60	21.01	54.80	20.92	- 2.23	0.04

Furthermore, as presented in Table 2, the average time to achieve the target blood pressure also differed significantly between the groups. Patients in the oral nifedipine group (Group A) achieved blood pressure control in an average of 45.60±21.01 minutes. Patients in the intravenous labetalol group (Group B) required a longer average time of 54.80±20.92 minutes. This observed difference in time to control blood pressure was statistically significant (p=0.04).

Complications

Table 3 details the incidence of complications observed between the two study groups. Neither group experienced hypotensive complications. Headache was reported in four patients in Group A and five patients in Group B; this difference was not statistically significant (p=1.00).

**Table 3:** Comparison of maternal complications between Group A (oral nifedipine) and Group B (intravenous labetalol) *p-value <0.05 statistically significant; chi-squared/Fisher's exact test applied

Complications	Groups		ꭓ^2^	P-value*
	Group A (%)	Group B (%)		
Hypotension				
Absent	50 (100)	50 (100)	-	-
Headache				
Present	4 (8)	5 (10)	-	Fisher's exact, 1.00
Absent	46 (92)	45 (90)		
Dizziness				
Present	3 (6)	12 (24)	6.35	0.03
Absent	47 (94)	38 (76)		
Palpitations				
Present	2 (4)	0 (0)	-	Fisher's exact, 0.49
Absent	48 (96)	50 (100)		
Tachycardia				
Present	12 (24)	0 (0)	13.63	0.001
Absent	38 (76)	50 (100)		
Nausea/vomiting				
Present	0 (0)	10 (20)	11.11	0.001
Absent	50 (100)	40 (80)		
Breathing difficulty				
Present	0 (0)	6 (12)	-	Fisher's exact, 0.03
Absent	50 (100)	44 (88)		
Bradycardia				
Present	0 (0)	8 (16)	-	Fisher's exact, 0.001
Absent	50 (100)	42 (84)		
Total	50 (100)	50 (100)	-	-

However, a statistically significant difference was observed in the incidence of dizziness, which was more prevalent in Group B (24%) compared to Group A (8%) (p=0.03). This suggests a higher association of dizziness with intravenous labetalol administration than with nifedipine. Palpitations occurred in two patients in Group A but in none in Group B, yet this association was not statistically significant (p=0.49).

Tachycardia was significantly more common in Group A, affecting 12 patients, while no patients in Group B experienced this complication (p=0.001). This finding indicates a stronger association between nifedipine administration and the occurrence of tachycardia. Nausea or vomiting was exclusively observed in Group B, affecting 10 patients, with no occurrences in Group A. This difference was statistically significant (p=0.03). Furthermore, breathing difficulty was noted in six patients and bradycardia in eight patients, both exclusively in Group B, with no cases in Group A. These associations were also statistically significant (p=0.03 and p=0.001, respectively).

Feto-maternal outcome

**Figure 1:**
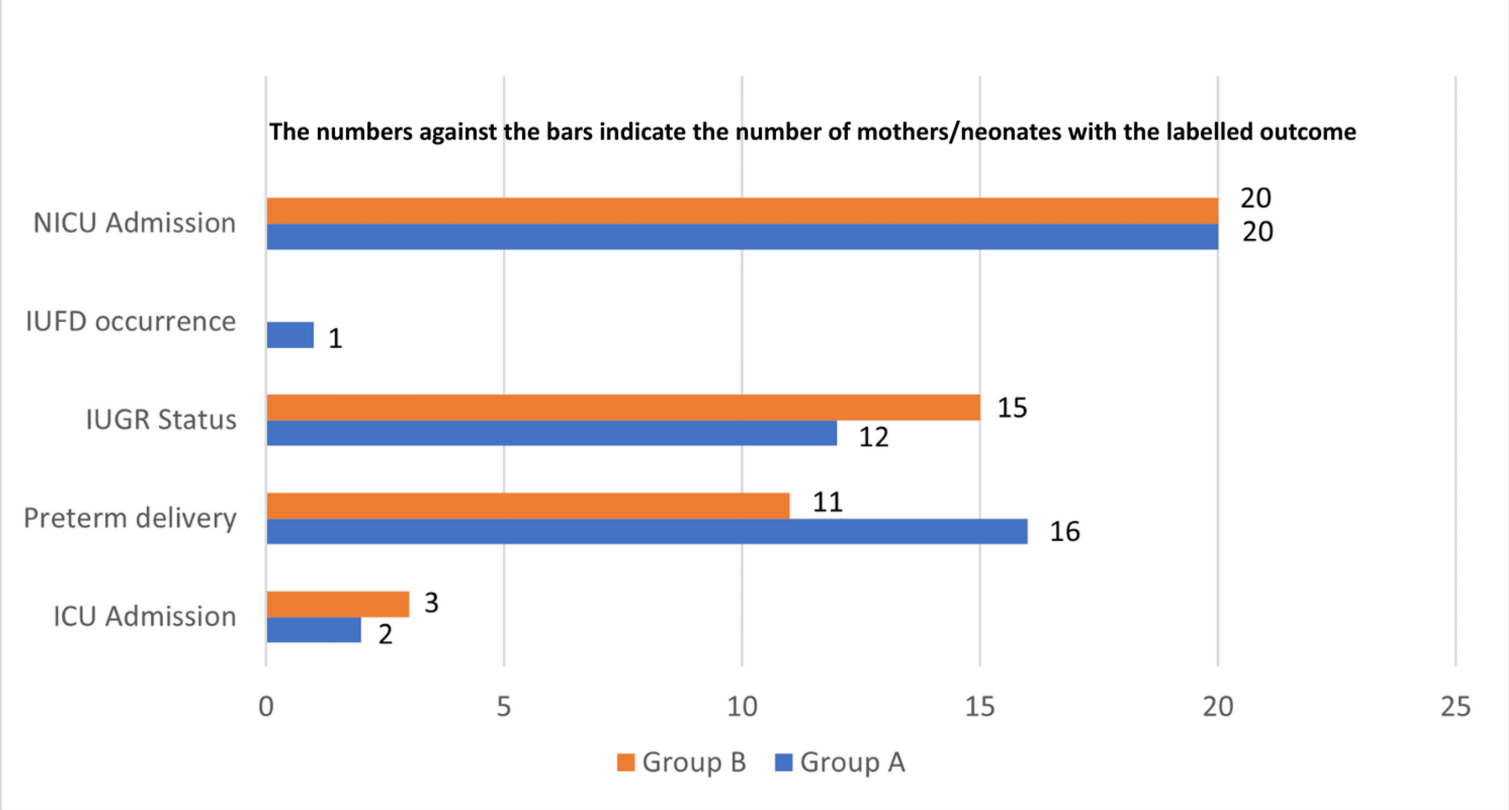
Comparison of feto-maternal outcome between Group A (oral nifedipine) and Group B (intravenous labetalol) NICU: neonatal intensive care unit; IUFD: intrauterine fetal demise; IUGR: intrauterine growth restriction; ICU: intensive care unit

Figure 1 provides a comparative analysis of feto-maternal outcomes between Group A and Group B. Post-delivery intensive care unit (ICU) admission was required for two patients in Group A and three patients in Group B; however, no statistically significant association was found between ICU admission and the type of drug administered (p=1.00).

Regarding gestational age at delivery (Figure 1), term deliveries occurred in 34 patients in Group A and 39 patients in Group B. Conversely, preterm deliveries were observed in 16 patients in Group A and 11 patients in Group B. Despite these variations, the association between the mode of delivery, the time of delivery status, and the type of drug administered was not statistically significant (p=0.26).

Intrauterine growth restriction (IUGR) was identified in 12 patients in Group A and 15 patients in Group B (Figure 1), but this difference was not statistically significant (p=0.50). One case of intrauterine fetal death (IUFD) occurred in Group A, with no such instances in Group B. No statistically significant association was found between birth status and the administered drug type (p=1.00). Finally, 20 neonates from each group required admission to the neonatal intensive care unit (NICU) following delivery, and no statistically significant association was found between NICU admission and the type of drug administered (p=1.00).

Cost-efficacy analysis

A significant difference in treatment cost was observed between the two groups. The mean cost incurred in the nifedipine group was ₹6.84±3.15, which was markedly lower than the mean cost of ₹1240±762.38 in the labetalol group. This disparity in cost between the two therapeutic approaches was statistically significant (p<0.001). The cost calculation exclusively accounted for the tablet cost of nifedipine and the injection cost of labetalol.

## Discussion

Baseline characteristics

The analysis of baseline characteristics revealed no statistically significant differences between the two study groups. Specifically, patient age distribution was comparable, with no significant difference observed (p=0.84). This finding aligns with a similar study by Biswas et al., which also reported comparable mean ages between nifedipine and labetalol groups (p=0.54) [6]. Kaur et al. similarly noted consistent mean ages of 25.85±3.10 years for the labetalol group and 24.92±3.12 years for the nifedipine group [7].

Regarding parity, Group A comprised 28 primigravida patients, while Group B included 30 primigravida patients, with no statistically significant difference between groups (p=0.68). These findings are consistent with observations by Lohnan et al., where 35 patients (47.3%) were nulliparous and the mean gestational age was 34.3±3.4 weeks, with 39 patients (52.7%) being 34 weeks and beyond [8].

Furthermore, vital parameters at admission, including mean age, mean weight, heart rate, and systolic and diastolic blood pressures, showed no statistically significant differences between the two groups (p>0.05). This comparability in baseline characteristics is corroborated by Biswas et al., who reported similar baseline vital parameters in both labetalol and nifedipine cohorts [6].

Efficacy

The study demonstrated significant differences in the efficacy of the two interventions. The mean number of doses required to achieve blood pressure control was lower in Group A (nifedipine group) at 2.28±1.05 doses compared to Group B (labetalol group) at 2.74±1.04 doses. This difference was statistically significant (p=0.03). Similarly, the average time to achieve target blood pressure was significantly shorter in the nifedipine group, requiring 45.60±21.01 minutes, versus 54.80±20.92 minutes in the intravenous labetalol group (p=0.04).

However, these findings contrast with those of Wasim et al., who reported comparable efficacy for both drugs in controlling blood pressure in patients with severe preeclampsia. Their study indicated that labetalol achieved target blood pressure in 22.6±13.5 minutes and nifedipine in 22.09±11.7 minutes (p>0.05), with similar dose requirements (labetalol: 2.3±1.58 doses; nifedipine: 2.2±1.58 doses; p>0.05) [9]. Shahnaz et al. also reported similar findings [10].

In alignment with our study, a systematic review by Lin et al. concluded that the nifedipine group required fewer doses to achieve target blood pressure (MD 0.62; 95% CI 0.36-0.88; p<0.00001) and achieved target blood pressure more rapidly (MD 7.64; 95% CI 4.08-11.20; p<0.0001) [11]. Kumari and Sinha similarly concluded that oral nifedipine controlled hypertension more rapidly and with fewer doses than intravenous labetalol [12]. Sahai et al. further supported that while both drugs are efficacious, nifedipine achieves more rapid hypertension control with fewer doses and is associated with a significant increase in urinary output [13].

Conversely, Kaur et al. found intravenous labetalol to be more rapid in controlling blood pressure in pregnant women with preeclampsia, suggesting its potential as a first-line agent for acute hypertensive emergencies [7]. Given these conflicting results, further research is warranted to definitively assess the superiority of either drug.

Complications

In the present study, headache was observed in four patients in Group A and five patients in Group B, with no statistically significant difference (p=1.00). However, dizziness was significantly more prevalent in the labetalol group (24%) compared to the nifedipine group (8%) (p=0.03). Palpitations were noted in two patients in Group A and none in Group B, a difference that was not statistically significant (p=0.49).

A statistically significant association was found between nifedipine administration and tachycardia, which occurred in 12 patients in Group A but in no patients in Group B (p=0.001). Conversely, nausea or vomiting was significantly more common in Group B, affecting 10 patients compared to none in Group A (p=0.03). Similarly, breathing difficulty (six patients) and bradycardia (eight patients) were exclusively observed in Group B and were statistically significant (p=0.03 and p=0.001, respectively).

No statistically significant association was found between ICU admission and the type of drug administered (p=1.00), with two patients from Group A and three from Group B requiring ICU admission. This outcome was primarily attributed to maternal complications of preeclampsia rather than drug effects. Biswas et al. similarly reported no statistically significant differences between groups for various maternal and neonatal complications, including ICU admission, maternal eclampsia, decreased maternal urine output, maternal heart failure, maternal stroke, maternal nausea and vomiting, maternal dizziness/headache, maternal palpitation, fetal heart rate abnormality, and five-minute Apgar score <7 [6]. Kaur et al. and Lin et al. also observed no significant differences in adverse effects between the two groups [7,11]. Consistent with these findings, Kumari and Sinha found no significant differences in side effects and feto-maternal outcomes [12].

Fetal outcome

Regarding fetal outcomes, IUGR was observed in 12 patients in Group A and 15 patients in Group B, but this difference was not statistically significant (p=0.50). One case of IUFD occurred in Group A, while no such cases were reported in Group B; however, no statistically significant association was found between birth status and the administered drug type (p=1.00).

Twenty neonates from each group were admitted to the NICU post-delivery, with no statistically significant association found between NICU admission and the type of drug administered (p=1.00). A systematic review by Lin et al. also reported no significant differences in neonatal complications, including NICU admission, five-minute Apgar score <7, neonatal deaths, and fetal heart rate abnormality [11].

Cost-effectiveness

The cost analysis revealed a substantial difference in treatment expenses between the two groups. The mean cost for the nifedipine group was ₹6.84, significantly lower than the mean cost of ₹1240 for the labetalol group (p=0.001). This finding is consistent with a study by Azmat et al., where the average treatment cost per participant in the intravenous labetalol group was $550 (₹46,200), while in the oral nifedipine group, it averaged $600 (₹50,400) [14]. This suggests that nifedipine may be more cost-effective than labetalol in this clinical context.

Limitations

This study has several limitations that may influence the generalizability and objectivity of its findings. Firstly, the single-center design coupled with a limited sample size may restrict the applicability of these results to a broader patient population. Secondly, the absence of randomization in the study design introduces the potential for observer bias, which could affect the objectivity of outcome assessments.

## Conclusions

This study, comparing oral nifedipine and intravenous labetalol for severe preeclampsia, found both to be effective first-line agents for blood pressure reduction. However, oral nifedipine demonstrated a quicker onset and required fewer doses, making it a convenient option, especially in settings with limited resources or challenging intravenous access. Conversely, intravenous labetalol offered predictable blood pressure control suitable for hospital management, despite having a higher incidence of certain side effects and comparable or greater time and dosing requirements than nifedipine. Ultimately, the choice between these medications should be individualized based on patient factors, clinical setting, and resource availability, with further research needed to validate these findings and explore long-term feto-maternal outcomes.
